# Differential Expression of miR-93 and miR-21 in Granulosa Cells and Follicular Fluid of Polycystic Ovary Syndrome Associating with Different Phenotypes

**DOI:** 10.1038/s41598-017-13250-1

**Published:** 2017-11-07

**Authors:** Mohammad Naji, Ashraf Aleyasin, Saeid Nekoonam, Ehsan Arefian, Reza Mahdian, Fardin Amidi

**Affiliations:** 1 Department of Anatomy, School of Medicine, Tehran University of Medical Sciences, Tehran, Iran; 2 Department of Infertility, Shariati Hospital, Tehran University of Medical Sciences, Tehran, Iran; 3 Molecular virology lab, Department of Microbiology, School of Biology, College of Science, University of Tehran, Tehran, Iran; 4 Pasteur Institute of Iran, Molecular Medicine Department, Tehran, Iran

## Abstract

The heterogeneous and multifactorial essence of polycystic ovary syndrome (PCOS) renders a remarkable significance to microRNAs (miRNAs). Normo-androgenic (NA) and hyperandrogenic (HA) PCOS patients were compared with matched healthy women. Expression of miRNAs and TGFβ signaling genes was studied by qRT-PCR and western blotting. Effect of androgen on expression of miR-93 and miR-21 and involvement of androgen receptor were appraised. In granulosa cells (GCs), miR-93 and miR-21 showed significantly increased levels in HA patients compared to NA patients. On the contrary, follicular fluid (FF) levels of both miRNAs were significantly decreased in HA group compared to control women. No significant change in the expression of miRNAs in serum samples was detected. Furthermore, mRNA levels of SMAD7 and TGFBR2 were significantly downregulated in GCs of HA group compared to NA and control subjects. TGFBR2 protein level was significantly decreased in HA patients compared to controls. Free testosterone and free androgen index were positively correlated with expression of miR-93 and miR-21 in GCs of PCOS group. Our findings show distinct molecular signature of different subtypes of PCOS. Intermediary position of miRNAs as androgen responsive factors may play critical role in the pathogenesis of PCOS in hyperandrogenic condition.

## Introduction

Polycystic ovary syndrome (PCOS) is a common and complex multifactorial endocrinopathy affecting 8–12% of reproductive-aged women^1^. PCOS manifests with a constellation of signs and symptoms such as androgen excess, ovulatory disturbance, polycystic ovaries and metabolic complications^2^. Aberrant folliculogenesis in PCOS is the leading cause of anovulatory infertility^3^. Increased proportion of growing follicles and impaired selection of dominant follicle, lead to arrest of folliculogenesis process at preantral stage^4^. Dysfunction of granulosa cells (GCs), as a result of disordered gene expression profile and secretory function, may contribute to the derangements of folliculogenesis in PCOS condition^5–7^. Androgen excess is the most prominent feature of PCOS which could be observed in 50–75% of women with PCOS^8^. Excessive androgen seems to be the “root cause” of PCOS and many associated complications of PCOS can be attributed to the elevated presence of androgens^9^. Although the source(s) of excessive androgen is unspecified, several studies have confirmed induction of PCOS-like phenotype including abnormal folliculogenesis by administration of exogenous androgens *in vivo* ^10–12^.

Members of transforming growth factor β (TGFβ) superfamily are well-studied signaling molecules with diverse and indisputable roles in developmental processes that affect multiple facets of folliculogenesis^13^. Knock-out (KO) models of TGFβ signaling mediators like SMAD2, SMAD3 and SMAD4 exhibited several defects in folliculogenesis, ovulation and fertility^14–16^. The importance of TGFβ signaling pathway and their regulatory molecules has been shown in pathogenesis of impaired follicular growth of PCOS^17–19^. MicroRNAs (miRNAs) are small noncoding RNA molecules composed of 20–24 nucleotides that post-transcriptionally regulate gene expression through interaction with mRNAs, particularly at 3′ untranslated region (3′-UTR), and modifying their stability or translation^20^. Dysregulation of miRNAs expression during the course of folliculogenesis could cause considerable complications and deviations in normal progression of the process. Conditional KO of Dicer-1 in cells expressing anti-Müllerian hormone receptor type 2 caused disordered folliculogenesis, and reduced ovulation rate and infertility^21,22^. Since miRNA’s machinery works via complementary sequences, hundreds of mRNAs could be targeted by a single miRNA molecule^23^. Consequently, a group of evolutionary conserved and functionally related genes such as TGFβ signaling members could be regulated by a few miRNAs. Accordingly, miRNAs targeting TGFβ members could play a significant role in pathologic conditions in which noticeable dysregulation of TGFβ signaling elements has been implicated.

To provide new insights into the pathogenesis and etiology of PCOS, miRNAs have received more attention from recent studies. In this regard, the expression profiles of miRNAs in circulation and follicular fluid (FF) of PCOS patients have been investigated to find novel biomarkers and target genes that may contribute in the diseased phenotype^24,25^. Recent studies has been reported the overexpression of miR-93 in circulation, adipose and ovarian tissues of PCOS patients^24,26,27^. It has been revealed that miR-93 could be an important factor in proliferative status of GCs^27^. In addition, it has been demonstrated that increased level of miR-21 in whole blood of PCOS patients has a correlation with obesity^28^. MiR-21 has been detected as the most abundant miRNA associated with RNA-induced silencing complex (RISC) in GCs, proposing its functional importance in GCs^29^. Moreover, miR-21 has been introduced as a critical player in apoptosis of GCs^30^ and regulation of adipogenic differentiation by affecting TGFβ signaling^31^. In consideration of GCs dysfunction in PCOS and the importance of TGFβ signaling in folliculogenesis; we hypothesized that androgen excess milieu in PCOS may deregulate miRNAs with multiple targets in TGFβ signaling pathway. In the current work, we studied the expression of miR-93 and miR-21 in GCs, FF and serum of normo-androgenic (NA) and hyperandrogenic (HA) PCOS patients. Moreover, relative expression of putative targets of miR-93 and miR-21 from TGFβ signaling pathway were investigated in GCs. We also studied the involvement of androgen receptor (AR) in mediation of androgen effects on miRNAs and downstream targets.

## Results

### Basic characteristics of PCOS and control participants

Demographic and clinical parameters of participants were listed in Table 1. No significant differences were observed in age, BMI, 17-OHP, FSH, LH/FSH ratio, E2, Prolactin, TSH, fasting Plasma glucose, fasting serum insulin and HOMA-IR values. Concentration of TT and FT were significantly increased in the serum of HA PCOS patients compared to NA PCOS patients and controls subjects. PCOS patients had significantly higher concentration of dehydroepiandrosterone sulfate (DHEAS). Ferriman-Gallwey score and FAI (free androgen index) were elevated in PCOS patients versus control subjects, and also in HA PCOS patients compared to NA PCOS patients. The concentrations of sex hormone-binding globulin (SHBG) and LH also were higher in HA PCOS patients.

**Table 1:** Basic clinical features of participants.

	Control (n = 25)	Normo-androgenic PCOS (n = 19)	Hyperandrogenic PCOS (n = 22)
Age	28.24 ± 0.82	28.89 ± 1.07	29 ± 0.66
BMI (kg/m^2^)	24.37 ± 0.73	25.94 ± 0.75	27.02 ± 0.97
Ferriman-Gallwey Score	2.8 ± 0.23	4.2 ± 0.24^a^	9.3 ± 0.78^b,c^
Total T (nmol/liter)	1.37 ± 0.14	1.96 ± 0.16	3.5 ± 0.24^b,c^
Free T (nmol/liter)	0.011 ± 0.001	0.015 ± 0.001	0.057 ± 0.018^b,c^
SHBG (nmol/liter)	61.22 ± 2.69	54.86 ± 4.37	45.26 ± 4.31^b^
FAI	2.26 ± 0.19	3.75 ± 2.8^a^	9.25 ± 1.15^b,c^
DHEAS (nmol/liter)	3414.21 ± 352.55	5102.03 ± 344.48^a^	6981.27 ± 629.12^b^
17-OHP (ng/ml)	0.96 ± 0.098	0.89 ± 0.106	1 ± 0.131
FSH (mIU/ml)	6.5 ± 0.36	5.81 ± 0.49	5.45 ± 0.41
LH (mIU/ml)	6.42 ± 0.44	8.27 ± 0.52	11.96 ± 1.51^b^
LH/FSH	1.05 ± 0.08	1.6 ± 0.15	2.46 ± 0.35
E2 (nmol/liter)	0.18 ± 0.012	0.16 ± 0.011	0.2 ± 0.01
Prolactin (ng/ml)	17.43 ± 1.26	14.84 ± 0.92	16.05 ± 1.52
TSH (µIU/ml)	2.26 ± 0.21	1.72 ± 0.15	2.01 ± 0.25
Fasting Plasma glucose (mmol/liter)	5.1 ± 0.14	5.29 ± 0.14	5.5 ± 0.2
Fasting serum insulin (mIU/liter)	5.26 ± 0.31	6 ± 0.43	6.34 ± 0.48
HOMA-IR	1.21 ± 0.08	1.41 ± 0.11	1.53 ± 0.12

^a^Significant difference between control and normo-androgenic subjects.

^b^Significant difference between control and hyperandrogenic subjects.

^c^Significant difference between normo-androgenic and hyperandrogenic subjects.

### Expression of miR-93 and miR-21 in granulosa cells, follicular fluid and serum

**Figure 1:**
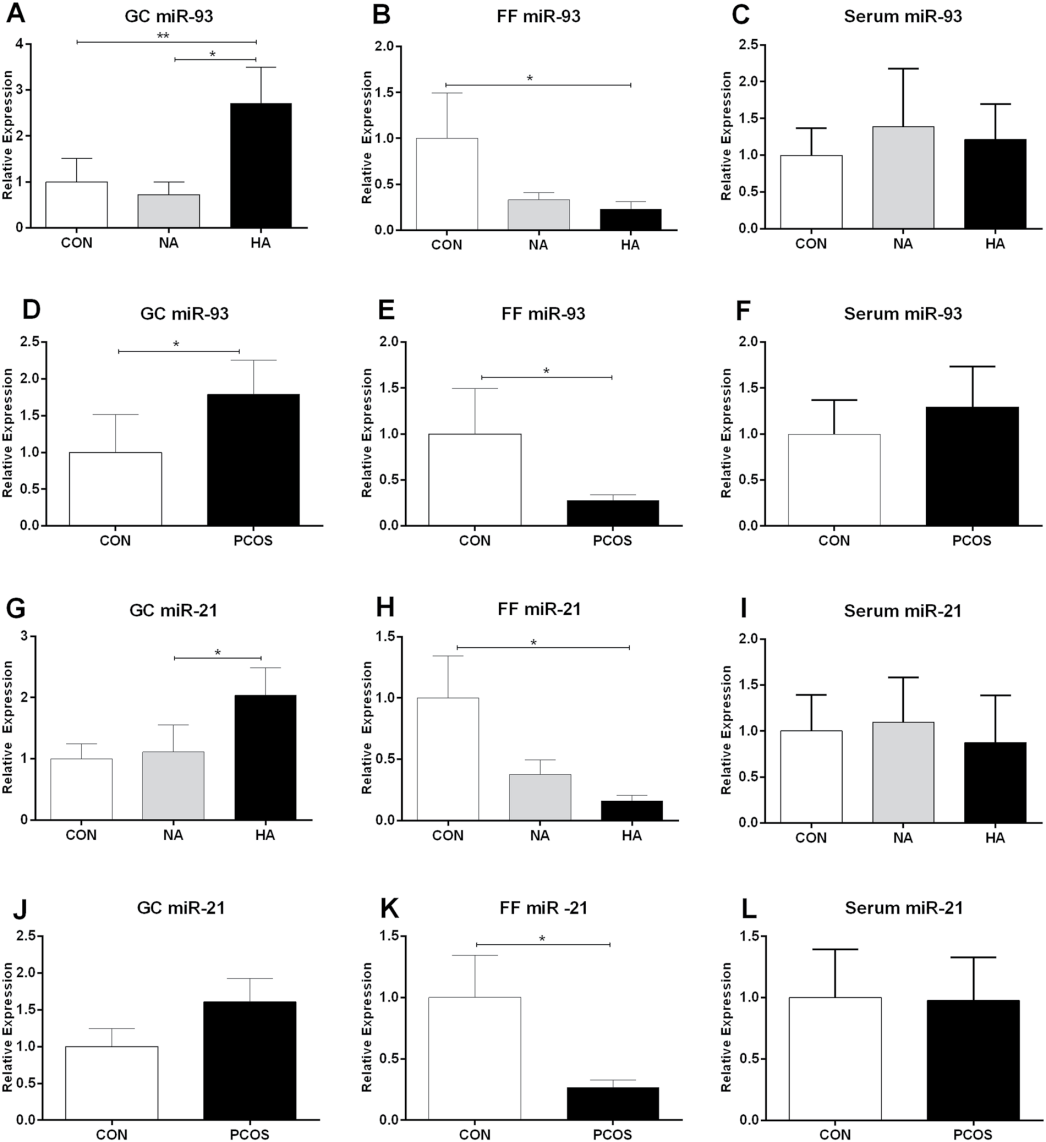
Relative expression of miR-93 and miR-21 in GCs, FF and serum. Expression levels of miR-93 in GCs (**A**), FF (**B**) and serum (**C**) of HA and NA PCOS patients were compared with control group (CON). (**D**–**F**) Expression levels of miR-93 were assessed after integration of PCOS patients in a single group. Relative expression levels of miR-21 in GCs (**G**), FF (**H**) and serum (**I**) of HA and NA PCOS patients were compared with CON group. (**J**–**L**) Relative expression of miR-21 was assessed after integration of PCOS patients into a single group. Relative expression levels of miRNAs were normalized against CON group. *P < 0.05, **P < 0.01.

Next we quantified the expression levels of miR-93 and miR-21 in GCs, FF and serum of human subjects. The expression level of miR-93 in GCs of HA PCOS patients was significantly upregulated compared to NA PCOS patients (*P* = 0.02) and control subjects (*P* = 0.004) (Fig. 1A). However, the expression level of miR-93 was significantly reduced in FF of HA PCOS patients in comparison to control group (*P* = 0.03) (Fig. 1B). Relative expression of miR-93 in serum did not indicate any significant alternation (Fig. 1C). Considering HA and NA PCOS patients as a single group of PCOS subjects, the expression of miR-93 was significantly higher in GCs (*P* = 0.03) of PCOS patients versus control subjects (Fig. 1D). Contrary to GCs, FF content of miR-93 was significantly reduced in PCOS group (*P* = 0.04) (Fig. 1E). Relative expression of miR-93 in serum of PCOS group was slightly upregulated but did not reach the significant threshold (Fig. 1F).

Relative expression of miR-21 in GCs were increased in HA PCOS patients when compared with NA PCOS (*P* = 0.01) (Fig. 1G). However, the expression level of miR-21 in FF of HA PCOS patients was significantly decreased compared to control subjects (*P* = 0.01) (Fig. 1H). No significant alteration in the relative expression of miR-21 in serum was detected (Fig. 1I). After aggregation of HA and NA PCOS patients, the relative expression of miR-21 in GCs was not altered (Fig. 1J). However, the relative expression of miR-21was downregulated in FF of PCOS patients (*P* = 0.01) (Fig. 1K). Serum content of miR-21 in PCOS group did not reveal any significant change (Fig. 1L).

### Expression of TGFβ signaling genes in granulosa cells

**Figure 2:**
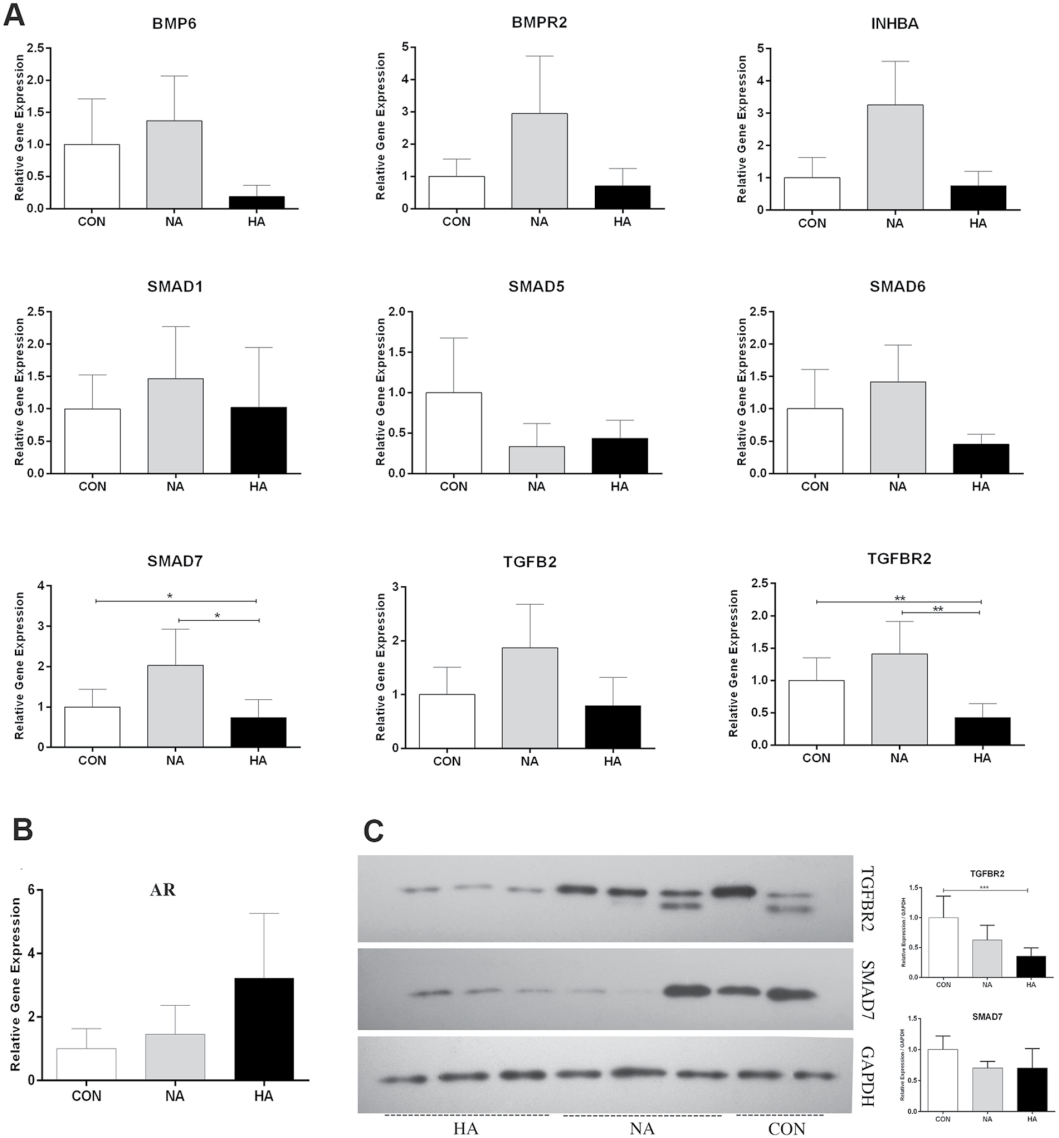
Expression of TGFβ signaling and AR genes in GCs of human subjects. (**A**) Significant decrease in the expression levels of SMAD7 and TGFBR2 in HA PCOS patients compared to NA PCOS patients and control subjects (CON). (**B**) No change in relative expression of AR was noticed between control subjects and both classes of PCOS patients. (**C**) Western blot experiments indicated reduced level of TGFBR2 (75 KDa) in HA group but no significant alteration in SMAD7 (46 KDa) was noticed. Expression of proteins was normalized against GAPDH (36 KDa). From right: lane 1 and 2, CON; lane 3-5, NA PCOS; lane 6-8, HA PCOS. *P < 0.05, **P < 0.01, ***P < 0.001.

We then quantified the expression levels of several key molecules of TGFβ signaling pathway in GCs. These TGFβ mediators were identified as presumptive targets of miR-93 and miR-21 with prominent roles in folliculogenesis. HA PCOS patients had significantly decreased expression levels of SMAD7 and TGFBR2 compared to NA PCOS patients (*P* = 0.02 and *P = *0.001, respectively). In addition, the expression levels of SMAD7 and TGFBR2 were also significantly reduced in HA PCOS patients versus control subjects (*P* = 0.04 and *P* = 0.002, respectively) (Fig. 2A). We did not observe any significant changes in relative expression of BMP6, BMPR2, INHBA, SMAD1, SMAD5, SMAD6 and TGFB2 (Fig. 2A). Also, there were no significant changes in relative expression of TGFβ signaling studied genes in PCOS group (HA and NA patients combined) versus control subjects (Supplementary Fig. S1A). Next, we quantified androgen receptor (AR) expression in GCs. HA PCOS patients had an increased level of AR expression but did not reach the significant threshold (Fig. 2B). We had similar observation even after integration of PCOS subjects in a single set (Supplementary Fig. S1B).

To confirm the gene expression results at the protein level, we analyzed the expression of TGFBR2 and SMAD7 by western blotting. Western blot analysis of GCs indicated a significant downregulation of TGFBR2 expression in HA PCOS group compared to control subjects (*P* = 0.001). There was no significant reduction of TGFBR2 expression in NA PCOS patients (Fig. 2C). Reduced expression of SMAD7 in HA and NA PCOS patients did not reach the significance threshold (Fig. 2C). Moreover, the expression of TGFBR2 was significantly diminished in PCOS group (HA and NA patients) when compared to control group (*P* = 0.002) (Supplementary Fig. S1C) but no significant change in SAMD7 alternation was observed (Supplementary Fig. S1D). Uncropped images of western blot films were illustrated in Supplementary Fig. S2.

### Correlation and ROC curve analysis

The results of correlation analysis were summarized in Supplementary Table S1. In all studied subjects, the expression of miR-93 and miR-21 in GCs were positively correlated with FT (ρ = 0.43, *P* = 0.0002; and ρ = 0.38, *P* = 0.002), while their expression in FF was negatively associated with FT concentration (ρ = −0.28, *P* = 0.02; and ρ = −0.31, *P* = 0.01, respectively). Furthermore, FAI was correlated with the expression of miR-93 in GCs (ρ = 0.3, *P* = 0.01) as well as miR-93 and miR-21 in FF (ρ = −0.26, *P* = 0.03; and ρ = −0.26, *P* = 0.03). Relative expression of miR-93 and miR-21 in FF showed inverse relationship with the levels of miR-93 and miR-21 in GCs (ρ = −0.28, *P* = 0.02; and ρ = −0.38, *P* = 0.002). Similarly, in PCOS group, miR-93 and miR-21 in FF were associated with GCs’ miRNAs (ρ = −0.33, *P* = 0.04; and ρ = −0.5, *P* = 0.001). In addition, the expression levels of miR-93 and miR-21 in GCs were positively correlated with FT (ρ = 0.39, *P* = 0.01; and ρ = 0.39, *P* = 0.01) and FAI (ρ = 0.35, *P* = 0.02; and ρ = 0.34, *P* = 0.02). TGFBR2 and SMAD7 protein levels were negatively correlated with TT and FT in all studied subjects and PCOS group.

In HA PCOS patients, the expression of miR-21 in GCs showed significant correlation with FAI (ρ = 0.43, *P* = 0.04) but there was an inverse correlation between miR-21 level in GCs and FF (ρ = 0.−49, *P* = 0.02). Protein level of SMAD7 in GCs of HA PCOS patients was negatively correlated with FT (ρ = −0.53, *P* = 0.032). Likewise, similar relationship between miR-21 expression in GCs and FF was observed in NA PCOS patients (ρ = 0.−4, *P* = 0.03). Furthermore, the correlations between the expression levels of miR-93 and miR-21 in GCs and FT concentration were significant and positive (ρ = 0. 5, *P* = 0.01; and ρ = 0.5, *P* = 0.01). In control group, TGFBR2 expression was negatively associated with TT and FAI (ρ = −0.51, *P* = 0.042; and ρ = −0.67, *P* = 0.007).

**Figure 3:**
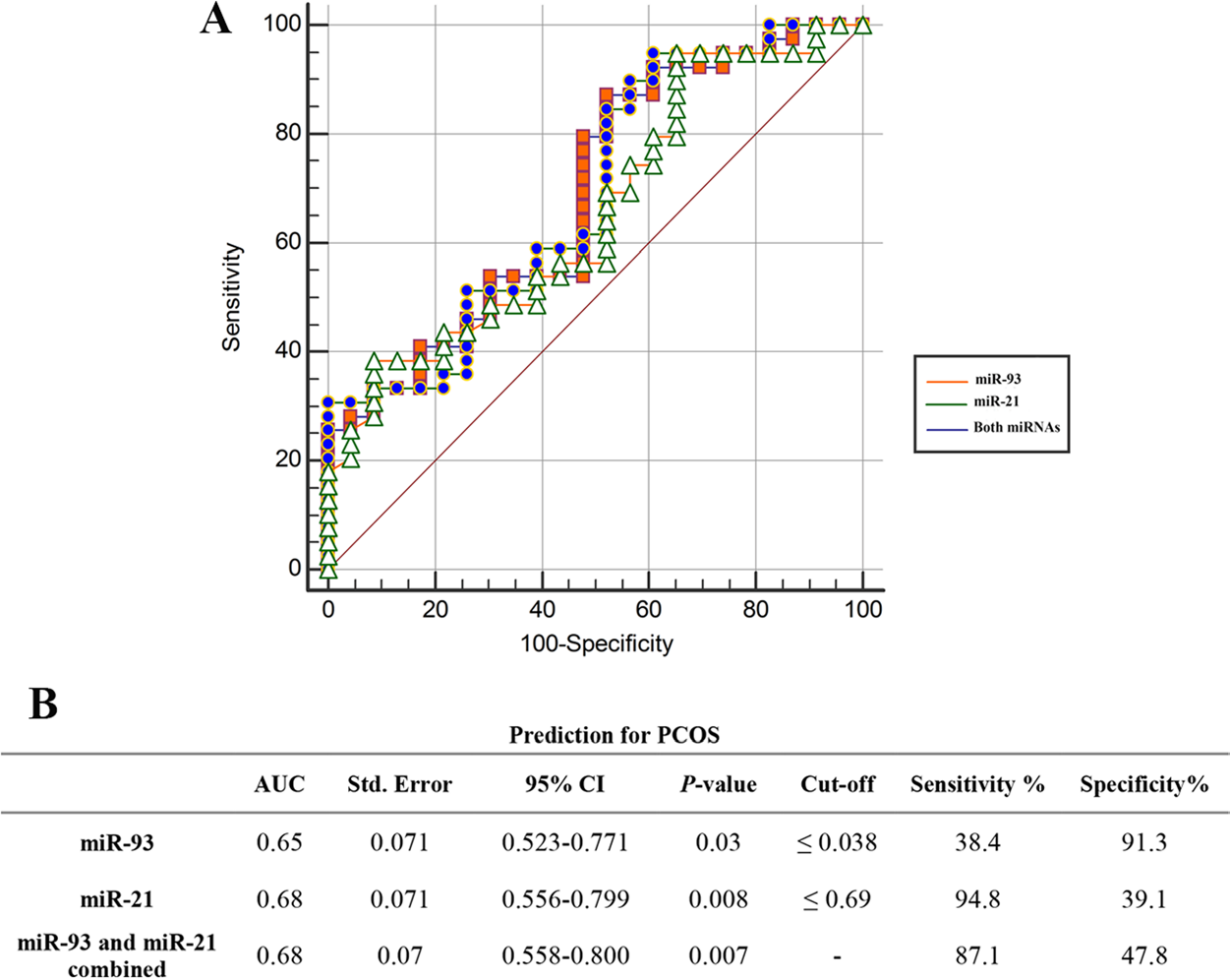
ROC curve analysis to determine the discriminative significance of follicular fluid levels of miR-93 and miR-21 for prediction of PCOS.

To evaluate the diagnostic value of differentially expressed miRNAs in FF for discrimination of PCOS patients from control subjects, ROC curves were plotted and the area under curve (AUC) was determined. MiR-21 had the largest AUC of 0.68 at cut-off values of 0.69, which yielded the highest Youden’s index, for discriminating PCOS patients from control subjects (Fig. 3A and B). Accordingly, miR-21 had a sensitivity and specificity of 94.8% and 39.1%, respectively. MiR-93 with AUC of 0.65 at cut-off values of 0.038 showed 38.4% sensitivity and 91.3% specificity (Fig. 3A and B). The combination of miRNAs did not improve the AUC of miR-21 (0.68) but increased the specificity to 47.8% and slightly reduced sensitivity to 87.1% which may offer a more potent diagnostic tool.

### Role of androgen and androgen receptor in regulation of miRNAs

**Figure 4:**
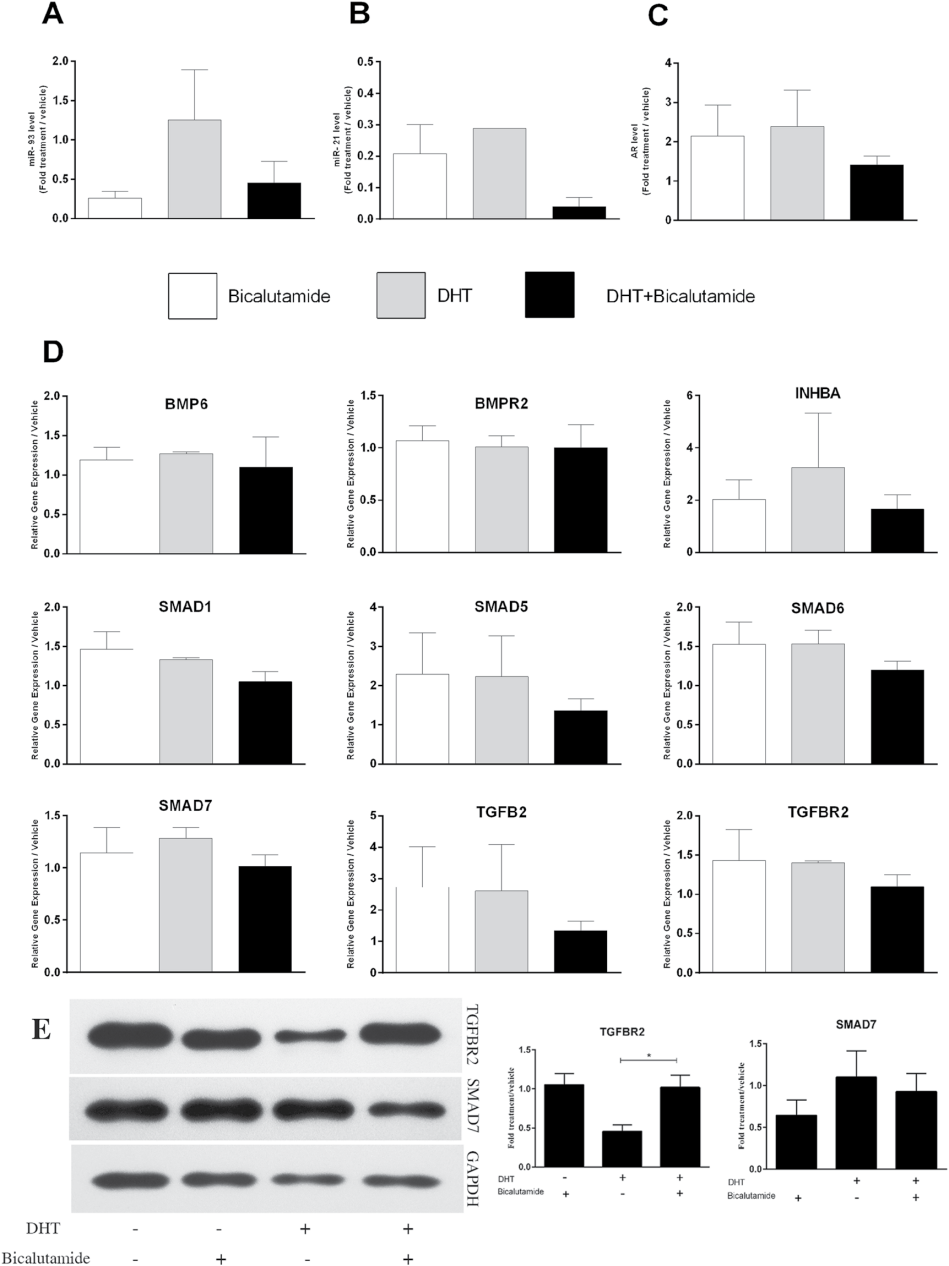
Role of androgen and AR in regulating the expression of miR-93, miR-21 and their putative targets from TGFβ signaling pathway. (**A**) Expression of miR-93 increased by DHT but AR blockade by bicalutamide treatment impeded DHT effect. (**B**) MiR-21 was slightly responded to DHT. (**C**) To demonstrate possible regulatory circuit between androgen and AR expression in GCs with intervention of AR as transcription factor, cultured GCs were treated with DHT, bicalutamide (AR blocker). DHT did not exert any effect on the expression of AR, nor bicalutamide was involved. (**D**) No element of TGFβ signaling pathway showed any specific pattern of expression which could be attributed to the elevated expression of miRNAs. (**E**) TGFBR2 protein expression was significantly decreased by DHT treatment while SMAD7 was not responsive to DHT. From left: lane 1, carrier (ethanol); lane 2, Bicalutamide; lane 3, DHT; lane 4, Bicalutamide and DHT. *P < 0.05.

To demonstrate the effect of androgen in upregulation of miR-93 and miR-21, and to elucidate the participation of AR in mediation of androgen effects on miRNAs; cultured GCs were treated with dihydrotestosterone (DHT) and bicalutamide. DHT treatment induced upregulation of miR-93 compared to bicalutamide, whereas pre-treatment of cells with bicalutamide blocked DHT effect on the expression of miR-93 (Fig. 4A). Viability evaluation by MTT assay revealed that different treatment regimens could not affect cell viability in comparison to vehicle, so the observed effect could not be attributed to side effects of treatment. The same pattern was also noticed for miR-21 but it was not as responsive as miR-93 to DHT stimulation (Fig. 4B). Stimulation of GCs with DHT and inhibition of AR by bicalutamide did not affect the expression level of AR gene (Fig. 4C). Androgen stimulation and AR blockade did not induce any significant pattern of expression in mRNAs of TGFβ signaling genes (Fig. 4D). However, analysis of protein expression showed significant downregulation of TGFBR2 by DHT treatment (*P = *0.04) (Fig. 4E). Different treatments did not induce any significant change in the expression level of SMAD7. Upstream sequences of transcription start site (5 Kb) of miR-93 and miR-21 were analyzed for presence of androgen response element (ARE), which indicated four ARE half sites (from −4737 to −2009) in miR-93 associated sequence and one well-conserved ARE (−3703 to −3689) for miR-21 (Supplementary Fig. S1E).

### Functional analysis of predicted targets of miR-93 and miR-21

**Figure 5:**
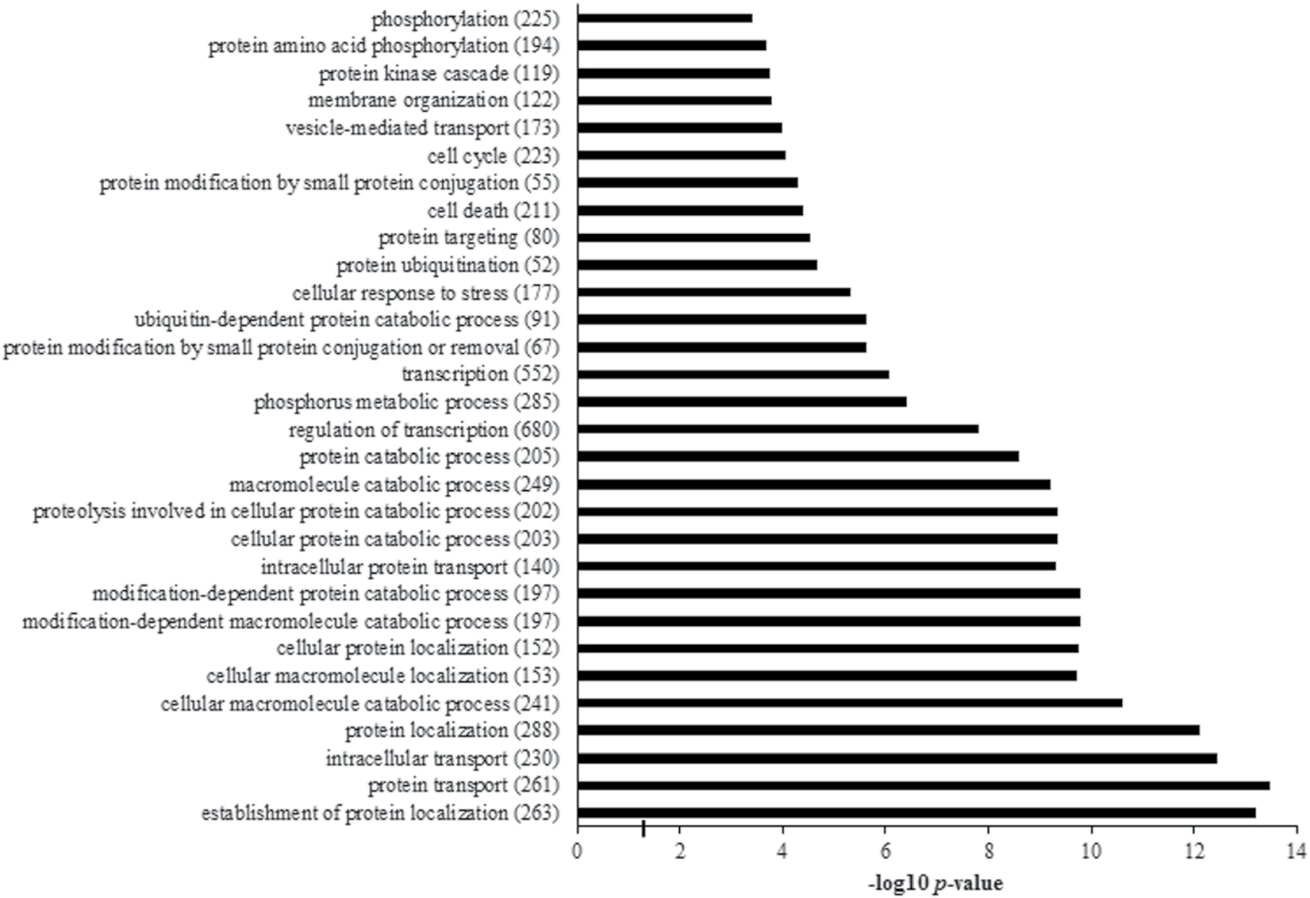
Top GO biological processes were identified by DAVID. Predicted targets of miR-93 and miR-21 after matching with granulosa expressing transcripts, were investigated for functional annotation. Benjamini-Hochberg adjusted *P*-values were depicted after −log10 transformation. Gene count in each enriched term was demonstrated in parenthesis.

To create a more realistic profile of predicted genes in context of GCs, a list of expressed transcripts of GCs from preovulatory follicles^32^ was utilized as a background. After integration of presumptive targets and exclusion of duplicate targets from TargetScan, miRanda, Microcosm, Mirwalk and miRDB, only common genes were selected to make lists of predicted targets which included 3324 genes for miR-93, 1414 genes for miR-21 and 3769 genes for miR-93 and miR-21 together. GO annotation for integrated target transcripts using DAVID identified several significant biological processes which could be categorized into five major classes after omission of obsolete and redundancy terms: (i) localization and transport, (ii) metabolic process, (iii) gene expression and RNA metabolic process, (iv) cell cycle and cell death and finally (v) signal transduction (Supplementary Table S2). Top 30 annotated biological processes are depicted in Fig. 5. GO analysis without incorporation of predicted targets of miR-93 and miR-21 identified a small number of new results (Supplementary Table S2).

**Figure 6:**
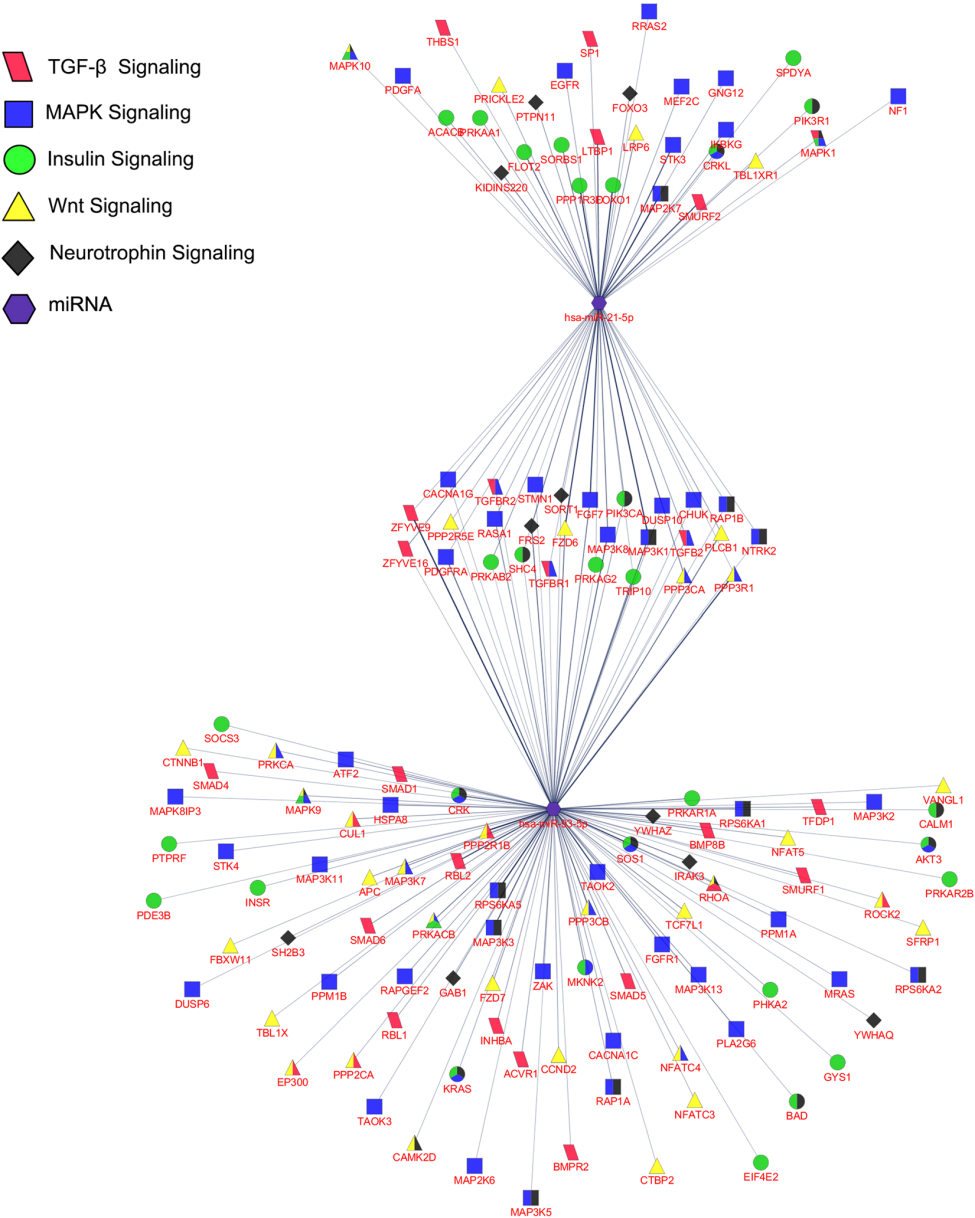
Network of miR-93 and miR-21 with putative target genes of important signaling pathways from KEGG enrichment analysis. The network was constructed by Cluepedia plugin in Cytoscape environment. Nodes reveal parallelogram for TGFβ signaling pathway, rectangle for MAPK signaling pathway, square for insulin signaling pathway, triangle for Wnt signaling pathway, diamond for neurotrophin signaling pathway, hexagon for miRNAs and edges for interactions.

KEGG pathway enrichment analysis of predicted targets revealed signaling pathways with important roles in folliculogenesis process and normal function of ovary including Neurotrophins, MAPK, Insulin, TGFβ and Wnt signaling pathways (Table 2). Enrichment analysis of predicted transcripts, individually targeted by miR-93 or miR-21, only identified prostate cancer pathway for miR-93. To illustrate the immense capability of a few miRNAs for targeting and modulating different signaling pathways, interactions of differentially expressed miRNAs and genes of above mentioned signaling pathways were networked (Fig. 6).

**Table 2:** KEGG pathway analysis of putative target genes for miR-93 and miR-21.

KEGG Pathways	Gene Count	P-value
Apoptosis	36	0.015
Colorectal cancer	35	0.0082
Pathways in cancer	100	0.019
Neurotrophin signaling pathway	45	0.015
Ubiquitin mediated proteolysis	45	0.019
Lysosome	42	0.022
MAPK signaling pathway	81	0.027
RNA degradation	24	0.029
Insulin signaling pathway	47	0.027
Endocytosis	59	0.025
Chronic myeloid leukemia	29	0.028
Pancreatic cancer	28	0.028
Adherens junction	29	0.037
Amino sugar and nucleotide sugar metabolism	19	0.046
Valine, leucine and isoleucine degradation	19	0.046
p53 signaling pathway	27	0.045
TGF-beta signaling pathway	31	0.053
Wnt signaling pathway	48	0.053

## Discussion

Scrutinizing the role and differential expression of miRNAs in various tissues of PCOS patients has been the subject of increasing number of studies^33^ which generally focused on miRNAs as potential biomarkers of the syndrome. The mechanism of miRNAs for modulation of gene expression, causes a broad range of signaling pathways and biological processes to be affected simultaneously when they are deregulated in pathological conditions. In the current study, we evaluated the expression levels of miR-93 and miR-21 in GCs, FF and serum of PCOS patients undergoing IVF cycles. Because of the significant role of androgen excess in many aspects of PCOS, patients were categorized into HA and NA groups to discover more precise link between different phenotypes of PCOS and expression of miRNAs. We found differential upregulation of miR-93 and miR-21 in GCs of HA PCOS patients. On the contrary, the expression levels of miR-93 and miR-21 in FF were significantly decreased in PCOS patients in comparison to control subjects. No significant difference in serum levels of miR-93 and miR-21 was noticed. Expression levels of predicted targets of miR-93 and miR-21 in TGFβ signaling pathway were also studied in GCs, and mRNA of SMAD7 and TGFBR2 were found to be significantly downregulated in HA PCOS patients. Moreover, TGFBR2 protein expression was significantly diminished in GCs of HA PCOS patients.

The essential role of androgen signaling in normal female reproductive function was proposed by defective folliculogenesis and fertility impairments of androgen receptor knockout (ARKO) mice^34^. Interestingly, conditional knockout of AR in GCs generally recapitulated the ARKO phenotype which reinforces the importance of AR signaling, especially through modulating the function of GCs in follicular development^35^. Moreover, a growing number of findings are clarifying the unquestionable involvement of miRNAs in folliculogenesis^21,22^. Seminal work of Sen *et al*. illustrated a functional circuit between androgen, miRNAs and folliculogenesis which miR-125b, as an androgen-responsive factor, attenuated follicular atresia^36^. In this study, we showed the elevated level of miR-93 in GCs of HA PCOS patients compared to NA PCOS patients and control subjects. Recently, miR-93 has been introduced to be a critical player in insulin resistance and proliferative status of GCs in PCOS via targeting GLUT4 in adipose tissue and CDKN1A in GCs, respectively^26,27^. It was also reported that PCOS patients presented higher level of miR-93 in circulation^24^. Furthermore, we showed overexpressed level of miR-21 in GCs of HA PCOS patients versus NA ones. Raised level of miR-21 in whole blood of PCOS patients^28^, its high association with RISC complex in GCs^29^ and involvement of miR-21 in apoptosis of GCs^30^ may indicate the significance of miR-21 in the pathogenesis of PCOS. The heterogeneous and multi-factorial features of PCOS, genetic background of subjects, disparity in inclusion criteria and different preparations of samples (plasma, serum and whole blood) may cause the inconsistency of our findings in serum with earlier reports^24,28^. The indispensable role of androgens in normal physiology of ovary and induction of PCOS-like phenotype^10–12^, and high incidence of androgen excess in PCOS patients; render the androgens a causative role in PCOS in which many accompanying complications are related^9^. The downstream androgen-responsive factors that mediate and diversify androgen’s effects on different organs remain to be fully characterized. MiRNAs can be potent and efficient androgen-responsive trans-acting factors in regulation of gene expression^36,37^. We demonstrated that human GCs in primary culture expressed AR and were responsive to DHT treatment which upregulated the expression of miR-93 and miR-21. Moreover, AR had direct role in the mediation of DHT effects on miRNAs as bicalutamide, an AR antagonist, inhibited the effects of DHT on the upregulation of miRNAs. Consistent with previous report regarding the presence of ARE in upstream sequence of miR-125b^36^, we found AREs in upstream sequences of miR-93 and miR-21. MiR-93 possesses multiple ARE half sites which have been reported to be efficient in AR binding^38^, while miR-21 has a single highly conserved ARE with well-defined and proved functionality for AR binding and promotion of miR-21 expression^39^. Contrary to the higher expression of miR-93 and miR-21 in GCs, these miRNAs were significantly decreased in FF of PCOS patients, especially in FF of HA PCOS patients. Intriguingly, expression of miRNAs in FF was negatively correlated with their counterparts in GCs. The exact sources of miRNAs in FF are unspecified but, intra‐ and extra‐ovarian origins are plausible due to close similarity between serum and FF components, especially those with low molecular masses^40,41^. The unchanged expression of miR-93 and miR-21 in serum and lack of correlation between the expression of miRNAs in serum and their counterparts in FF, may reflect the significance of intra‐ovarian factors in dysregulation of miR-93 and miR-21. Expression level of miRNAs in FF reflects the overall alternations in any diseased condition; rather than GCs which are the place of active transcription and function of miRNAs. In addition, upregulation of miRNAs in the GCs does not inevitably end in the same pattern of changes in FF, as the uncharacterized process of secretion and the large size of FF pool connected with circulation would be influential.

TGFβ signaling pathway possesses an integral role in normal physiology and function of ovary^13–16^. It was identified that a majority of TGFβ signaling related genes were deregulated in PCOS cumulus cells, including TGFβ receptors and SMADs^42^. This pathway could be also involved in fetal origin of PCOS, while in the meantime reinforced the importance of androgen in PCOS^43^. It has been experimentally verified that SMAD7 is a direct target of miR-93 and miR-21, and through these interactions, TGFβ signal transduction can be controlled^44,45^. Smad7 has been introduced as a key mediator of TGFβ signaling in normal function of GCs and oocyte-somatic cell interaction^46^. Dysregulation of Smad7 may result in impaired follicle development due to its important participation in apoptosis of GCs^47^.

In addition, Liu *et al*. showed that miRNAs could regulate the function of Smad7 in apoptosis of GCs^48^. It has also been observed that TGFBR2 could be targeted and inhibited by miR-93 and miR-21^31,37,49^. DHT treatment in ovarian cancer cells decreased the expression level of TGFBR2^50^. In this line, Mishra *et al*. using prostate cancer model showed that miR-21 is an androgen-responsive miRNA that could attenuate the expression of TGFBR2, and AR is the key mediator of androgen effects on the expression of miR-21^37^. Effective targeting of a receptor by a miRNA could modulate signaling of a pathway and elicit vast downstream consequences such as miR-21 contribution in adipogenic differentiation through targeting of TGFBR2^31^.

Classification of significant GO terms revealed that up/downregulation of a small number of miRNAs, owing to their mode of action, may spread massive modifications in homeostasis of biological systems. KEGG analysis enriched the presumptive targets of miR-93 and miR-21 into important signaling pathways apart from TGFβ signaling. Among all the enriched pathways, neurotrophin^51^, MAPK^52,53^, insulin^54^ and Wnt^55^ signaling pathways have been recognized to play critical roles in normal ovarian functions and folliculogenesis. FOXO3 plays significant role in folliculogenesis process and its phosphorylation status has been reported to be regulated by androgen via phosphatidylinositol 3-kinase/Akt pathway^56^. In silico analysis indicated that FOXO3 is a plausible target of miR-21 which may propose more complex regulatory network in PCOS through androgen effects on miRNAs adjusting the activity of transcription factors. Samples of the current study and many preceding works, were harvested after stimulation protocols for IVF treatment which may resolve many impeding elements in disturbed follicle development. Consequently, the observed differences could be very important as the stimulation protocols were not able to entirely reverse them. Taken together, regulation of miRNAs by numerous factors in conjunction with their extensive mechanism of gene targeting create puzzling networks of regulatory determinants with substantial role in diseased phenotypes such as PCOS. Additional in-depth studies are required to find the detailed functional importance of miRNAs in the development and pathophysiology of PCOS. Molecular dissimilarities between different subclasses of PCOS, as reinforced in this study, may reflect unlike etiological mechanisms and explain the inconsistent findings of studies which have tried to introduce miRNAs as potential biomarkers of PCOS.

## Methods

### Human subjects

This study was approved by the Research Deputy and Ethics Committee of Tehran University of Medical Sciences, and all subjects were enrolled at the Infertility Department of Shariati Hospital (Tehran, Iran). All participants signed an informed consent before enrollment. All methods were performed in accordance with guidelines and protocols of Tehran University of Medical Sciences. PCOS patients were diagnosed according to the revised Rotterdam European Society of Human Reproduction and Embryology/American Society for Reproductive Medicine Criteria^57^ which necessitates the incidence of at least two of the following criteria: 1) signs of clinical and/or biochemical hyperandrogenism, 2) oligo and/or anovulation, and 3) polycystic ovaries. The PCOS patients were divided into normo-androgenic (NA, n = 19) and hyperandrogenic (HA, n = 22) groups based on free testosterone (FT) level of serum (cutoff, 0.024 nmol/L). In addition, NA patients had normal circulating levels of total testosterone (TT), FT, DHEAS and no symptoms of hyperandrogenism, like hirsutism, androgenic alopecia, and acne. Control subjects (n = 25) were selected for *in vitro* fertilization (IVF) due to male factor infertility and sex selection. They had regular periods, no clinical or biochemical sign of androgen excess and no background of menstrual irregularities. All subjects underwent their first cycle of IVF and did not take any medication interfering with sex hormone secretion, lipid and glucose metabolism at least three months before the study. Women with history and evidence of endocrinological diseases, premature ovarian insufficiency and endometriosis were excluded.

Basic clinical features of participants were assessed by recording body mass index (BMI) and Ferriman-Gallwey score, and measurement of fasting serum levels of TT, FT, sex hormone-binding globulin (SHBG), DHEAS, FSH, LH, estradiol, 17-Hydroxyprogesterone (17-OHP), prolactin, thyroid-stimulating hormone (TSH), glucose and insulin. Free androgen index (FAI) was calculated as testosterone × 100/SHBG (nmol/L). Homeostasis model assessment (HOMA) was utilized to appraise the degree of insulin resistance (IR).

### Controlled ovarian hyperstimulation

The same controlled ovarian hyperstimulation procedure was applied in all subjects by GnRH antagonist protocol. Briefly, administration of recombinant FSH (Gonal-F, Serono, Italy; 150-300 IU) was started at day three of menstrual cycle and followed until two follicles with diameter of 14-15 mm were observed. Dosages of gonadotropins were adjusted according to the patient’s age, estradiol levels and transvaginal ultrasonic measurements of the follicles. Subsequently, GnRH antagonist (cetrorelix; ASTA Medica, Amsterdam, The Netherlands; 0.25 mg/day) was administrated and sustained until the observation of at least two follicles with the diameter of 18 mm. Finally, Oocyte retrieval was carried out 36 hours after administration of 10,000 IU human chorionic gonadotropin (hCG) (Choriomon, IBSA Institut Biochimique S.A., Switzerland).

### Collection of granulosa cells, follicular fluid and serum samples

Mural GCs were isolated from follicular aspirates by cell strainer technique. Briefly, aspirates were passed through 40 µm cell strainer (BD Biosciences, CA, USA) and retained clusters of GCs were harvested by back-wash of the strainer with PBS containing 1% BSA (Bovine serum albumin; Sigma, Louis, MO, USA). Clusters were dispersed by repeatedly pipetting, followed by filtering through 70 µm cell strainer to exclude undispersed aggregates from final cell suspension. Blood contamination of cell suspension was monitored by presence of CD45 expressing cells via flow cytometry. Finally, cell suspensions were centrifuged for 5 min at 1500 × g in 4 °C and cell pellets were snap frozen in liquid nitrogen and stored at −80 °C for further experiments. FF of the first punctured follicles during pickup procedure was collected and centrifuged for 5 min at 1500 × g in 4 °C to exclude cells and tissue fragments. The supernatant was stored at −80 °C for RNA isolation. Serum samples were also prepared as FF samples and stored at −80 °C for further experiments.

### Culture of granulosa cells

To culture GCs, follicular aspirates from three normal cycling women were pooled and processed as mentioned above under sterile condition. Cells were resuspended in Dulbecco’s Modified Eagle Medium/F12 (DMEM/F12) supplemented with 10% fetal Bovine Serum (FBS) (Biowest, France) and 1% penicillin/streptomycin (Biowest, France). Cells were plated in T-75 culture flask and incubated in humidified atmosphere with 5% CO_2_ at 37 °C. The growing medium was refreshed every other day until sufficient number of cells were obtained. For treatment of the cells, they were detached and plated (1500/cm^2^) in 6-well plates for 48 hours. Afterward, cells were starved for 48 hours by replacing the growing medium with DMEM/F12 supplemented with 10% charcoal stripped FBS (Sigma, Louis, MO, USA). Cells were treated in four groups: (i) vehicle (ethanol) (ii) vehicle and 25 nM of DHT (Sigma, Louis, MO, USA) (iii) vehicle and 30 µM bicalutamide, AR antagonist, (Santa Cruz Biotechnology, Inc, Santa Cruz, CA) and (iv) bicalutamide and DHT (bicalutamide was added 2 hours before DHT). Cells were treated for 24 hours before RNA and protein extraction. For western blotting experiments, cells were cultured and treated in 10-cm dishes. All results were normalized against vehicle group.

### Quantitative real-time PCR

Total RNA was extracted from GCs was using TRIzol reagent (Thermo Scientific, Waltham, MA, USA) according to manufacturer instruction. For extraction of RNA from FF and serum, 200 µl of each sample was used. RNA was quantified by WPA spectrophotometer (Biochrom) and genomic DNA contamination was removed using DNase I (RNase-free) (Thermo Scientific, Waltham, MA, USA). Reverse transcription was performed by RevertAid first-strand cDNA synthesis kit (Thermo Scientific, Waltham, MA, USA); random hexamer and stem-loop RT primer (0.375 µM) were used for cDNA synthesis of mRNAs and miRNAs, respectively. All primers and probes were designed by AlleleID 6 software (Supplementary Tables S3 and S4) and synthesized by Macrogen (Macrogen, South Korea). For quantification of mRNAs, components of PCR reactions (20 µl) were as follows: 10 μl 2X RealQ Plus MasterMix Green (Ampliqon, Denmark), 0.8 μl of each primer, 2 μl first-strand cDNA template (1:6 in distilled water) and 6.4 μl distilled water. Thermocycling parameters were 15 min at 95 °C for enzyme activation, and 35 cycles of 95 °C for 20 seconds followed by 60 °C for 60 seconds using Rotor-Gene Q instrument (Qiagen). To quantify miRNAs, each reaction (20 μl) consisted of 10 μl 2X RealQ Plus MasterMix for Probe (Ampliqon, Denmark), 0.8 μl of each primer, 0.5 μl probe, 2 μl first-strand cDNA template (1:8 in distilled water) and 5.9 μl distilled water. Thermocycling was conducted as follows: 95 °C for 15 min to activate enzyme, 40 cycles of 95 °C for 25 seconds followed by 60 °C for 60 seconds. Expression levels of miRNAs and mRNAs were normalized against expression of RNU6-1 RNA (U6)^58–60^ and GAPDH, respectively. Using 2^−∆Ct^ method, relative expression of genes was calculated.

### Western blot analysis of SMAD7 and TGFBR2

Cells were lysed in RIPA buffer containing protease and phosphatase inhibitors (50 mM Beta-glycerophosphate, 1 mM PMSF, 5 µg/ml Leupeptin, 10 µg/ml Pepstatin A, 1 mM EDTA, 5 mM EGTA, 10 mM NaF, 1 mM Sodium orthovanadate), and protein concentration was quantified by Bradford solution. Protein extracts (20 µg) were resolved in 12% sodium dodecylsulfate polyacrylamide gels (SDS–PAGE) and then transferred to polyvinylidene difluoride (PVDF) membranes. Membranes were blocked in 1% casein solution at 4 °C overnight followed by incubation in mouse monoclonal anti SMAD7 (1:300, Santa Cruz Biotechnology, CA, USA), mouse monoclonal anti TGFBR2 (1:3000, Santa Cruz Biotechnology, CA, USA) and mouse monoclonal anti GAPDH (1:1000, Abcam, Cambridge, MA, USA). Afterward, primary antibodies were probed by incubation in horse radish peroxidase (HRP)-conjugated anti mouse IgG secondary antibody (1:3000, Santa Cruz Biotechnology, CA, USA) for 1 hour at room temperature. Membranes were incubated in ECL reagent (ECL-plus, Thermo Scientific, Waltham, MA, USA) and then exposed to western blotting films (Thermo Scientific, Waltham, MA, USA). The intensity of bands was quantified using ImageJ sofware (NIH, Bethesda, MD, USA) and normalized against GAPDH intensity. Western blotting was performed for 16 controls, 17 NA and 15 HA PCOS patients. Cell culture experiments were repeated four times, using cells from independent isolations.

### Bioinformatic analysis

Putative targets of miR-93 and miR-21 were predicted by five algorithms: TargetScan (http://www.targetscan.org), miRanda (http://www.microrna.org), Microcosm (http://www.ebi.ac.uk/enright-srv/microcosm/htdocs/targets/v5/), Mirwalk (http://zmf.umm.uni-heidelberg.de/apps/zmf/mirwalk2/) and miRDB (http://www.mirdb.org/miRDB/). TGFβ signaling pathway genes with important role in folliculogenesis were selected for quantification alongside the miRNAs (BMP6, BMPR2, INHBA, SMAD1, SMAD5, SMAD6, SMAD7, TGFB2 and TGFBR2). In addition, the integrated list of putative targets of both miRNAs was compared with a transcriptome dataset of mural GCs of preovulatory follicles^32^. Only common predicted targets were further used for gene ontology (GO) annotation and KEGG (Kyoto Encyclopedia of Genes and Genomes) pathway enrichment analysis utilizing Database for Annotation, Visualization and Integrated Discovery (DAVID) (https://david.ncifcrf.gov). Benjamini-Hochberg multiple testing correction was used to adjust *P*-values. Cytoscape (version 3.4.0) was used to make network of the miRNAs with members of important signaling pathways in KEGG pathway analysis. Upstream sequences of miR-93 and miR-21 were analyzed for presence of androgen response elements (ARE). To this end, 5 Kb upstream of transcription start site was scanned for presence of canonical ARE (5′-AGAACAnnnTGTTCT-3′) and ARE half site (5′-AGAACA-3′) using FIMO tool of MEME suite (4.11.2) with *P*-value < 0.001^61^.

### Statistical analysis

Normally distributed data (Age, BMI, SHBG, 17-OHP, FSH, E2, Prolactin, TSH, fasting plasma glucose, fasting serum insulin, HOMA-IR) and non-normally distributed data (mFG, TT, FT, FAI, DHEAS, LH, LH/FSH, real-time PCR and western blot results) were analyzed by ANOVA or Kruskal–Wallis tests, respectively. Tukey’s and Dunn’s multiple comparison tests were applied after ANOVA and Kruskal–Wallis tests, respectively. Correlation of miRNAs in GCs with miRNAs in FF and SMAD7 and TGFBR2; and between expression of genes and TT, FT, DHEAS and FAI was evaluated by Spearman’s rank correlation. Receiver operating characteristic (ROC) analysis for expression of miRNAs in FF was performed by MedCalc software, combination of miR-93 and miR-21 for ROC analysis was carried out using multiple logistic regression. All data were presented as mean ± standard error of mean (SEM) and *p*-values < 0.05 were considered as significant. SPSS 22 (SPSS Inc., Chicago, IL) and GraphPad prism were used for statistical analysis.

## Electronic supplementary material

Supplementary materials
