# Supplementary material for: Differential Expression of miR-93 and miR-21 in Granulosa Cells and Follicular Fluid of Polycystic Ovary Syndrome Associating with Different Phenotypes
Source: Sci Rep. 2017 Nov 7;7:14671. doi: 10.1038/s41598-017-13250-1 (PMC5676684; doi:10.1038/s41598-017-13250-1)
Supplement: Supplementary file 1 — Supplementary materials [file 41598_2017_13250_MOESM1_ESM.pdf]

Differential Expression of miR-93 and miR-21 in Granulosa Cells and Follicular Fluid of Polycystic Ovary Syndrome Associating with Different Phenotypes

Mohammad Naji[a], Ashraf Aleyasin[b], Saeid Nekoonam[c], Ehsan Arefian[d], Reza Mahdian[e], Fardin Amidi[f]*.

[a]Department of Anatomy, School of Medicine, Tehran University of Medical Sciences, Tehran, Iran. Email: naji_m_f@yahoo.com

[b]Department of Infertility, Shariati Hospital, Tehran University of Medical Sciences, Tehran, Iran. Email: ivfshariati69@gmail.com

[c]Department of Anatomy, School of Medicine, Tehran University of Medical Sciences, Tehran, Iran. Email: nekoonam_saeid@yahoo.com

[d]Molecular virology lab, Department of Microbiology, School of Biology, College of Science, University of Tehran, Tehran, Iran. Email: arefian@gmail.com

[e]Pasteur Institute of Iran, Molecular Medicine Department, Tehran, Iran. Email: dr.reza.mahdian@gmail.com

*[f]Department of Anatomy, School of Medicine, Tehran University of Medical Sciences, Tehran, Iran. Email: Famidi@sina.tums.ac.ir

***Correspondence and reprint request:** Fardin Amidi. Department of Anatomy, School of Medicine, Tehran University of Medical Sciences, Pour Sina St, Tehran, Iran. Postal code: 1417613151. Tell/Fax: (+98) 9123188556/ (+98) 21- 8898 9487. Email: Famidi@sina.tums.ac.ir

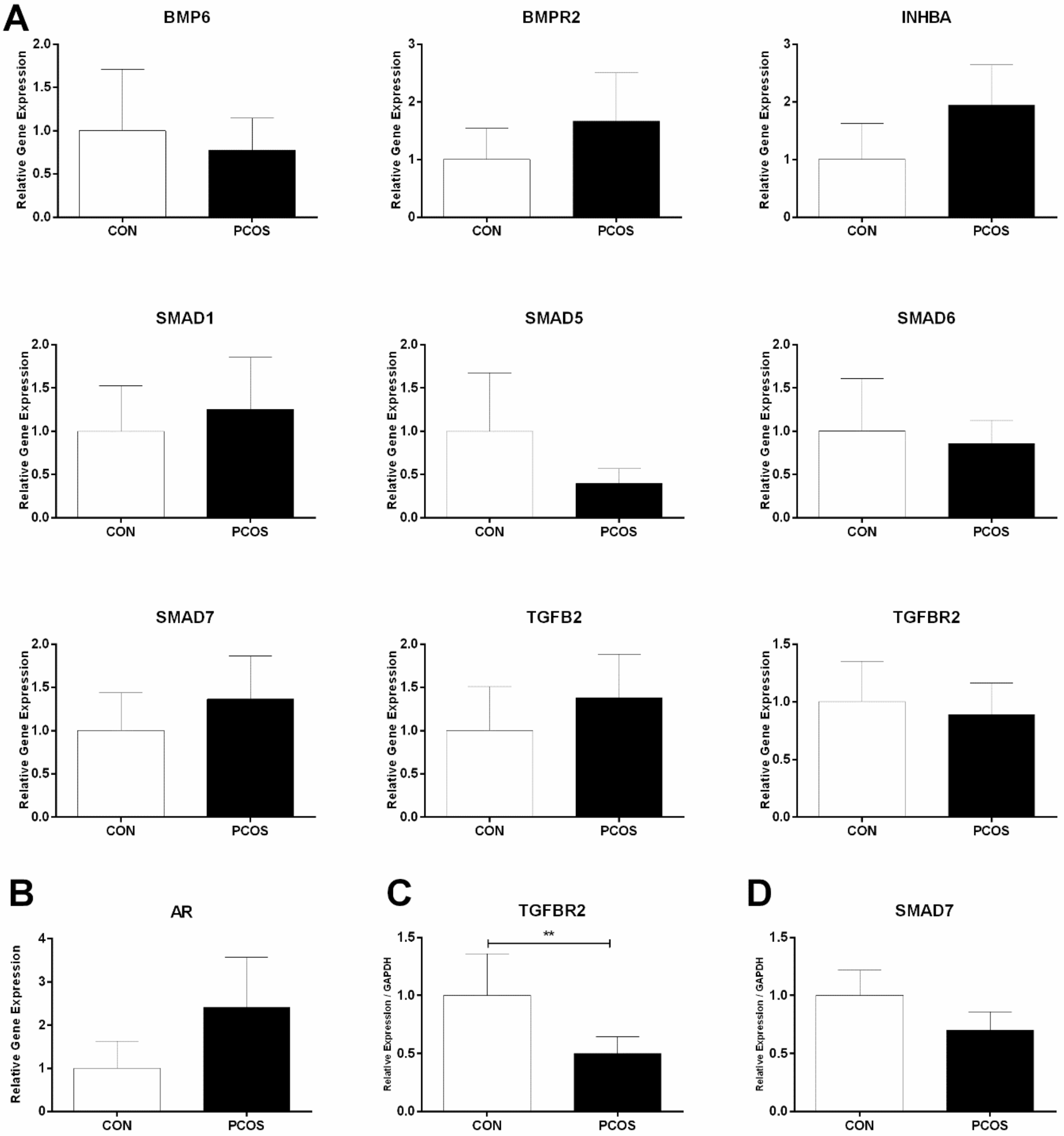

E

miR-93
ARE half site (5´-AGAACA-3´)
(-4737) AGAACA (-4732)
(-4644) AGAACA (-4639)
(-4385) AGAACA (-4380)
(-2014) AGAACA (-2009)

miR-21
ARE consensus sequence (5´-AGAACAnnnTGTTCT-3´)
(-3703) AGAACAagcTGTTAC (-3689)

Supplementary Fig S1

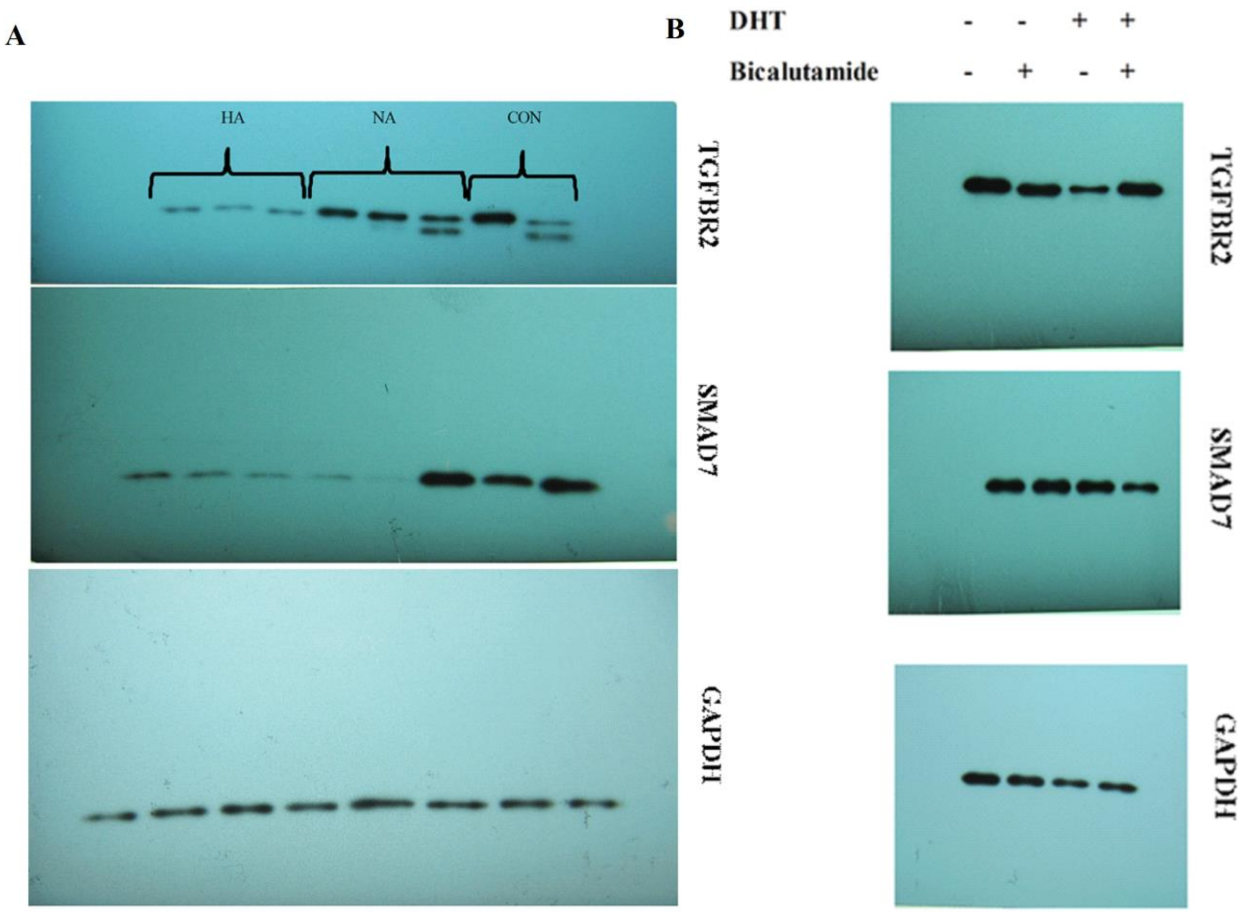

Supplementary Fig S2

Supplemental Figure Legend

Supplementary Figure 1. Expression of TGFβ signaling genes, AR and proteins of TGBR2 and SMAD7 in granulosa cells of PCOS and control subjects. (A) There was no significant difference in the relative expression of TGFβ signaling elements in PCOS subjects compared to control ones. (B) After integration of PCOS patients into a single group, AR expression did not reveal any alternation relative to control group. (C) TGFBR2 expression in GCs of PCOS patients was significantly lowered but (D) diminished level of SMAD7 was not significant. (E) Upstream sequences (5 Kb) of miR-93 and miR-21 were explored. Four ARE half sites for miR-93 and one well-conserved ARE for miR-21 were detected.

Supplementary Figure 2. Uncropped images of western blot experiment. (A) Western blotting of human granulosa cells. From right: lane 1 and 2, control samples (CON); lane 3-5, normo-androgenic (NA) PCOS; lane 6-8, hyperandrogenic (HA). (B) Western blotting of human granulosa cells after treatment with androgen and Bicalutamide. From left: lane 1, carrier (ethanol); lane 2, Bicalutamide; lane 3, DHT; lane 4, Bicalutamide and DHT.

Supplementary Table S1. Correlation analysis between androgen levels and expression of miRNAs, SMAD7 and TGFBR2.

**All subjects**			
		**ρ**	***P* value**
**FT**	GC miR-93	0.43	0.0002
**FT**	GC miR-21	0.38	0.002
**FT**	FF miR-93	-0.28	0.02
**FT**	FF miR-21	-0.31	0.01
**FAI**	GC miR-93	0.3	0.01
**FAI**	FF miR-93	-0.26	0.03
**FAI**	FF miR-21	-0.26	0.03
**GC miR-93**	FF miR-93	-0.28	0.02
**GC miR-21**	FF miR-21	-0.38	0.002
**GC miR-93**	TGFBR2 mRNA	0.26	0.04
**TT**	TGFBR2 protein	-0.53	0.008
**FT**	TGFBR2 protein	-0.52	0.0014
**FAI**	TGFBR2 protein	-0.53	0.009
**DHEAS**	TGFBR2 protein	-0.37	0.008
**TT**	SMAD7 protein	-0.36	0.01
**FT**	SMAD7 protein	-0.29	0.039
**PCOS**			
		**ρ**	***P* value**
**FT**	GC miR-93	0.39	0.01
**FT**	GC miR-21	0.39	0.01
**FAI**	GC miR-93	0.35	0.02
**FAI**	GC miR-21	0.34	0.02
**DHEA-S**	TGFBR2 mRNA	0.46	0.003
**GC miR-93**	FF miR-93	-0.33	0.04
**GC miR-21**	FF miR-21	-0.5	0.001
**TT**	TGFBR2 protein	-0.37	0.033
**FT**	TGFBR2 protein	-0.38	0.031
**TT**	SMAD7 protein	-0.5	0.003
**FT**	SMAD7 protein	-0.38	0.025
**Hyperandrogenic PCOS**			
		**ρ**	***P* value**
**FAI**	GC miR-21	0.43	0.04
**GC miR-21**	FF miR-21	-0.49	0.02
**FT**	SMAD7 protein	-0.53	0.032
**Normo-androgenic PCOS**			
		**ρ**	***P* value**
**FT**	GC miR-93	0.5	0.01
**FT**	GC miR-21	0.5	0.01
**GC miR-21**	FF miR-21	-0.4	0.03
**Control**			
		**ρ**	***P* value**
**TT**	TGFBR2 protein	-0.51	0.042
**FAI**	TGFBR2 protein	-0.67	0.007

Supplementary Table S2. Categorized annotated biological processes

	Biological Process	Gene Count	Benjamini adjusted P value
1	establishment of protein localization	263	6.1E-14
2	protein transport	261	3.3E-14
3	intracellular transport	230	3.6E-13
4	protein localization	288	8E-13
5	cellular macromolecule localization	153	1.9E-10
6	cellular protein localization	152	1.8E-10
7	intracellular protein transport	140	4.7E-10
8	protein targeting	80	0.00003
9	vesicle-mediated transport	173	0.0001
10	protein import	50	0.002
11	protein localization in organelle	53	0.0046
12	Golgi vesicle transport	48	0.0071
13	endocytosis	69	0.025
14	protein import into nucleus	32	0.051
15	cellular macromolecule catabolic process	241	2.4E-11
16	modification-dependent macromolecule catabolic process	197	1.6E-10
17	modification-dependent protein catabolic process	197	1.6E-10
18	cellular protein catabolic process	203	4.4E-10
19	proteolysis involved in cellular protein catabolic process	202	4.6E-10
20	macromolecule catabolic process	249	6.3E-10
21	protein catabolic process	205	2.5E-09
22	phosphorus metabolic process	285	0.00000038
23	protein modification by small protein conjugation or removal	67	0.0000023
24	ubiquitin-dependent protein catabolic process	91	0.0000024
25	protein ubiquitination	52	0.000022
26	protein modification by small protein conjugation	55	0.000051
27	protein phosphorylation	194	0.0002
28	phosphorylation	225	0.00038
29	positive regulation of macromolecule metabolic process	237	0.00072
30	negative regulation of macromolecule metabolic process	206	0.0011
31	positive regulation of nitrogen compound metabolic process	183	0.0016
32	regulation of cellular protein metabolic process	140	0.002
33	positive regulation of nucleobase, nucleoside, nucleotide and nucleic acid metabolic process	177	0.002
34	negative regulation of nitrogen compound metabolic process	151	0.0021
35	negative regulation of nucleobase, nucleoside, nucleotide and nucleic acid metabolic process	147	0.0051
36	negative regulation of cellular biosynthetic process	159	0.005
37	negative regulation of macromolecule biosynthetic process	155	0.0061
38	negative regulation of biosynthetic process	161	0.0069
39	proteolysis	273	0.011
40	dephosphorylation	53	0.014
41	positive regulation of biosynthetic process	187	0.015
42	protein dephosphorylation	47	0.015
43	positive regulation of cellular biosynthetic process	184	0.018

44	regulation of kinase activity	104	0.022
45	regulation of protein modification process †	80	0.039
46	nucleoside metabolic process	26	0.043
47	regulation of protein kinase activity	99	0.045
48	negative regulation of kinase activity	33	0.055
49	negative regulation of protein kinase activity †	30	0.056
50	lipid modification	27	0.057
51	regulation of transcription	680	0.000000016
52	transcription, DNA-templated	552	0.00000086
53	negative regulation of gene expression	151	0.00055
54	regulation of transcription from RNA polymerase II promoter	205	0.00085
55	negative regulation of transcription	137	0.0017
56	positive regulation of RNA metabolic process	138	0.0076
57	positive regulation of transcription, DNA-dependent	137	0.0075
58	positive regulation of transcription	158	0.0082
59	regulation of RNA metabolic process	449	0.0083
60	positive regulation of gene expression	162	0.0082
61	negative regulation of transcription, DNA-dependent	106	0.01
62	negative regulation of RNA metabolic process	107	0.012
63	regulation of transcription, DNA-dependent	437	0.014
64	RNA biosynthetic process	89	0.02
65	positive regulation of macromolecule biosynthetic process	176	0.022
66	translation	96	0.038
67	RNA processing ‡	71	0.043
68	positive regulation of transcription from RNA polymerase II promoter	105	0.05
69	cell death	211	0.000041
70	cell cycle	223	0.00009
71	regulation of apoptosis	222	0.0015
72	regulation of cell death	224	0.0018
73	regulation of programmed cell death	223	0.0019
74	apoptotic process	172	0.0019
75	cell cycle process	160	0.0051
76	programmed cell death	174	0.002
77	negative regulation of apoptosis	108	0.0044
78	negative regulation of cell death	109	0.0052
79	negative regulation of programmed cell death	108	0.0069
80	cell cycle arrest †	37	0.01
81	positive regulation of programmed cell death	125	0.011
82	positive regulation of apoptosis	124	0.012
83	mitotic cell cycle	109	0.012
84	positive regulation of cell death	125	0.013
85	G1/S transition of mitotic cell cycle	25	0.014
86	induction of programmed cell death	96	0.015
87	regulation of neuron apoptotic process	34	0.029
88	negative regulation of neuron apoptotic process	22	0.046
89	protein kinase cascade	119	0.00018
90	enzyme linked receptor protein signaling pathway	105	0.0044
91	MAPKKK cascade	62	0.0087

92	intracellular signaling cascade	320	0.011
93	negative regulation of MAP kinase activity	18	0.024
94	stress-activated protein kinase signaling pathway	26	0.034
95	small GTPase mediated signal transduction †	82	0.042
96	transmembrane receptor protein serine/threonine kinase signaling pathway	37	0.044
97	cellular response to stress	177	0.0000046
98	membrane organization	122	0.00017
99	negative regulation of cellular component organization	51	0.0073
100	response to DNA damage stimulus	111	0.008
101	heart development	68	0.023
102	chromatin organization	109	0.023
103	membrane invagination	69	0.025
104	negative regulation of molecular function	97	0.035
105	vasculature development	76	0.038
106	regulation of cellular response to stress	37	0.044
107	vasculogenesis †	18	0.044
108	response to metal ion	44	0.045
109	blood vessel development	74	0.046
110	response to cytokine stimulus	30	0.052
111	chromosome organization	132	0.058

Localization&Transport

Metabolic Process

Gene Expression and RNA metabolic Process

Cell Cycle and Cell Death

Signal Transduction

Miscellaneous

† Annotated biological process from hsa-mir-93-5p presumptive targets which was not coexisted in annotated processes of common targets of both microRNAs.

‡ Annotated biological process from hsa-mir-21-5p presumptive targets which was not coexisted in annotated processes of common targets of both microRNAs.

Supplementary Table S3. Sequences of primer pairs

BMP6	Forward	5′AGTCTTACAGGAGCATCAGC3′
	Reverse	5′ATTCCAGCCAGCCTTCTTC3′
BMPR2	Forward	5′AACACCACTCAGTCCACCTC3′
	Reverse	5′GTCTCCTGTCAACATTCTGTATCC3′
INHBA	Forward	5′GAGAACGGGTATGTGGAGATAG3′
	Reverse	5′GGTCACTGCCTTCCTTGG3′
SMAD1	Forward	5′GCTCAGTTATTGGCACAGTC3′
	Reverse	5′CCTGGCGGTGGTATTCTG3′
SMAD5	Forward	5′GATTCTGCTTGGGTTTGTTGTC3′
	Reverse	5′AATATGCTGCTGTCACTGAGG3′
SMAD6	Forward	5′CCGAATCTCCGCCACCTC3′
	Reverse	5′GTAGCCTCCGTTTCAGTGTAAG3′
SMAD7	Forward	5′AGATGCTGTGCCTTCCTC3′
	Reverse	5′GTCTTCTCCTCCCAGTATGC3′
TGFB2	Forward	5′TCAGACACTCAGCACAGCAG3′
	Reverse	5′GCAGCAAGGAGAAGCAGATG3′
TGFBR2	Forward	5′GACTTTCTTCATGTGTTCCTGTAG3′
	Reverse	5′TTGCTGGTGTTATATTCTTCTGAG3′
AR	Forward	5′TTGTCCATCTTGTCGTCTTCG3′
	Reverse	5′GCCTCTCCTTCCTCCTGTAG3′
GAPDH	Forward	5′GCTTCGCTCTCTGCTCCTC3′
	Reverse	5′CGACCAAATCCGTTGACTCC3′

Supplementary Table S4. Sequence of oligonucleotides for quantification of miRNAs.

**hsa-mir-21-5p (5′-3′)**	
RT-Primer	GTCGTATCCAGTGCAGGGTCCGAGGTATTCGCACTGGATACGACTCAACA
Forward Primer	GCCCTAGCTTATCAGACTG
Reverse Primer	TGCAGGGTCCGAGGTA
Taq-Man Probe	FAM-TGTTGAGTCGTATCCAGTGCG-BHQ1
**hsa-mir-93-5p (5′-3′)**	
RT-Primer	GTCGTATCCAGTGCAGGGTCCGAGGTATTCGCACTGGATACGACCTACCT
Forward Primer	GGGCAAAGTGCTGTTCG
Reverse Primer	TGCAGGGTCCGAGGTA
Taq-Man Probe	FAM-CAGGTAGGTCGTATCCAGTGCG-BHQ1
**U6 (5′-3′)**	
Forward Primer	GCTTCGGCAGCACATATAC
Reverse Primer	ATTTGCGTGTCATCCTTGC
Taq-Man Probe	FAM-CAGG**G**GCCATGCTAATCTTCTCT-BHQ1

G was replaced by deoxyinosine during synthesis
